# Machine QA for the Elekta Unity system: A Report from the Elekta MR‐linac consortium

**DOI:** 10.1002/mp.14764

**Published:** 2021-04-01

**Authors:** David A. Roberts, Carlos Sandin, Panu T. Vesanen, Hannah Lee, Ian M. Hanson, Simeon Nill, Thijs Perik, Seng Boh Lim, Sastry Vedam, Jinzhong Yang, Simon W. Woodings, Jochem W. H. Wolthaus, Brian Keller, Geoff Budgell, Xinfeng Chen, X. Allen Li

**Affiliations:** ^1^ Elekta Limited Cornerstone London Road Crawley RH10 9BL United Kingdom; ^2^ Philips Healthcare Vantaa Finland; ^3^ Allegheny Health Network Cancer Institute Pennsylvania USA; ^4^ The Joint Department of Physics The Institute of Cancer Research and The Royal Marsden NHS Foundation Trust UK; ^5^ Department of Radiation Oncology The Netherlands Cancer Institute–Antoni van Leeuwenhoek Hospital Amsterdam the Netherlands; ^6^ Memorial Sloan Kettering Cancer Center New York USA; ^7^ Department of Radiation Physics The University of Texas MD Anderson Cancer Center Texas USA; ^8^ Department of Radiotherapy University Medical Center Utrecht Utrecht the Netherlands; ^9^ Odette Cancer Centre Sunnybrook Health Sciences Centre Toronto Ontario Canada; ^10^ Christie Medical Physics and Engineering The Christie NHS Foundation Trust Wilmslow Road Manchester United Kingdom; ^11^ Department of Radiation Oncology Froedtert Hospital and Medical College of Wisconsin Milwaukee USA

**Keywords:** radiotherapy, quality assurance, MRI‐linac, dosimetry

## Abstract

Over the last few years, magnetic resonance image‐guided radiotherapy systems have been introduced into the clinic, allowing for daily online plan adaption. While quality assurance (QA) is similar to conventional radiotherapy systems, there is a need to introduce or modify measurement techniques. As yet, there is no consensus guidance on the QA equipment and test requirements for such systems. Therefore, this report provides an overview of QA equipment and techniques for mechanical, dosimetric, and imaging performance of such systems and recommendation of the QA procedures, particularly for a 1.5T MR‐linac device. An overview of the system design and considerations for QA measurements, particularly the effect of the machine geometry and magnetic field on the radiation beam measurements is given. The effect of the magnetic field on measurement equipment and methods is reviewed to provide a foundation for interpreting measurement results and devising appropriate methods. And lastly, a consensus overview of recommended QA, appropriate methods, and tolerances is provided based on conventional QA protocols. The aim of this consensus work was to provide a foundation for QA protocols, comparative studies of system performance, and for future development of QA protocols and measurement methods.

## 1 INTRODUCTION

The use of magnetic resonance imaging (MRI) in radiotherapy (RT) is becoming more commonplace, not only for simulation (MR‐Sim), but also as a component of treatment workflows with the introduction of integrated MRI and Linac systems (MR‐linac).^1^, ^2^, ^3^, ^4^, ^5^, ^6^ With new technology and workflows comes the need to assess how quality assurance (QA) from conventional practice is applied and whether new tests need to be introduced. This drives the need for an assessment of the test equipment and QA methods. The MR‐linac devices also introduce new online adaptive workflows for which traditional QA is no longer adequate or may not be practical due to modification of the treatment plan immediately prior to radiation delivery.

While there are numerous guidelines for machine QA on conventional systems,^7^, ^8^, ^9^ no consensus on QA for MR‐linac devices has yet been published. Several publications have considered individual aspects of QA on MR‐linac devices. Measurement equipment performance,^10^, ^11^, ^12^, ^13^, ^14^, ^15^, ^16^, ^17^, ^18^ reference^19^ and relative^15^, ^20^, ^21^, ^22^, ^23^ dosimetry within the presence of a magnetic field, treatment planning system,^24^, ^25^, ^26^, ^27^, ^28^ Linac,^22^, ^29^, ^30^, ^31^ MR device commissioning,^32^, ^33^ and routine QA^32^, ^34^, ^35^, ^36^, ^37^ are the broad range of areas addressed. While not considerably different to conventional RT QA, there are several aspects that need considering. Ensuring correct measurements, particularly relating to the effect of magnetic field on dosimeters and the alignment of the MR and MV coordinate systems are two key areas.

This report is an output of Elekta’s MR‐linac consortium^38^ which is an international collaboration of institutions and manufacturers (Elekta and Philips) to develop the MR‐linac clinical protocols and technology. A QA working group forms part of this consortium, with its remit to identify, develop, and review QA methods. The group also communicates the QA methods, equipment, and results to aid in the adoption of MR‐linac technology in the clinic. This report has been developed by the authors, through reviewing current conventional QA practice, literature review of MR‐linac research, and through the authors’ institutions implementation of QA protocols on clinical MR‐linac systems. The authors of this report formed a QA working group subcommittee to compile and review this guidance document with the following charges:

- To review the machine QA for an MR‐linac, specifically Elekta Unity, as compared to those required for a conventional RT system and provide guidance for QA tests.
- To provide an overview of the key aspects that need to be considered when performing measurements within a magnetic field.
- To provide a QA framework for comparative studies of system performance, enabling future development of QA protocols and measurement methods.

The report, as stated, focuses on the longitudinal QA measurements. Measurements conducted during commissioning^31^, ^39^ or after a service activity are not covered in this report. However, it is expected that many of the tests will be appropriate after a service operation and should be chosen based on the activity performed and based on manufacturer’s recommendations. Reference/adaptive plan QA and end‐to‐end QA equipment are identified but the reader is referred to other publications for testing techniques.^4^, ^29^, ^34^, ^40^, ^41^, ^42^, ^43^

While many of the comments and recommendations will be applicable to other configurations of MR‐linac devices, the authors are working with Elekta Unity systems, and thus the tests are designed around this system.

The recommendations within this report are intended to give an overview of suitable tests and methods and do not replace the manufacturer instructions for use or other labeling provided with the device. Individual institutions should assess their QA needs based on their own regulations and risk analysis as appropriate.

### 1.1 Elekta Unity overview

**Fig. 1:**
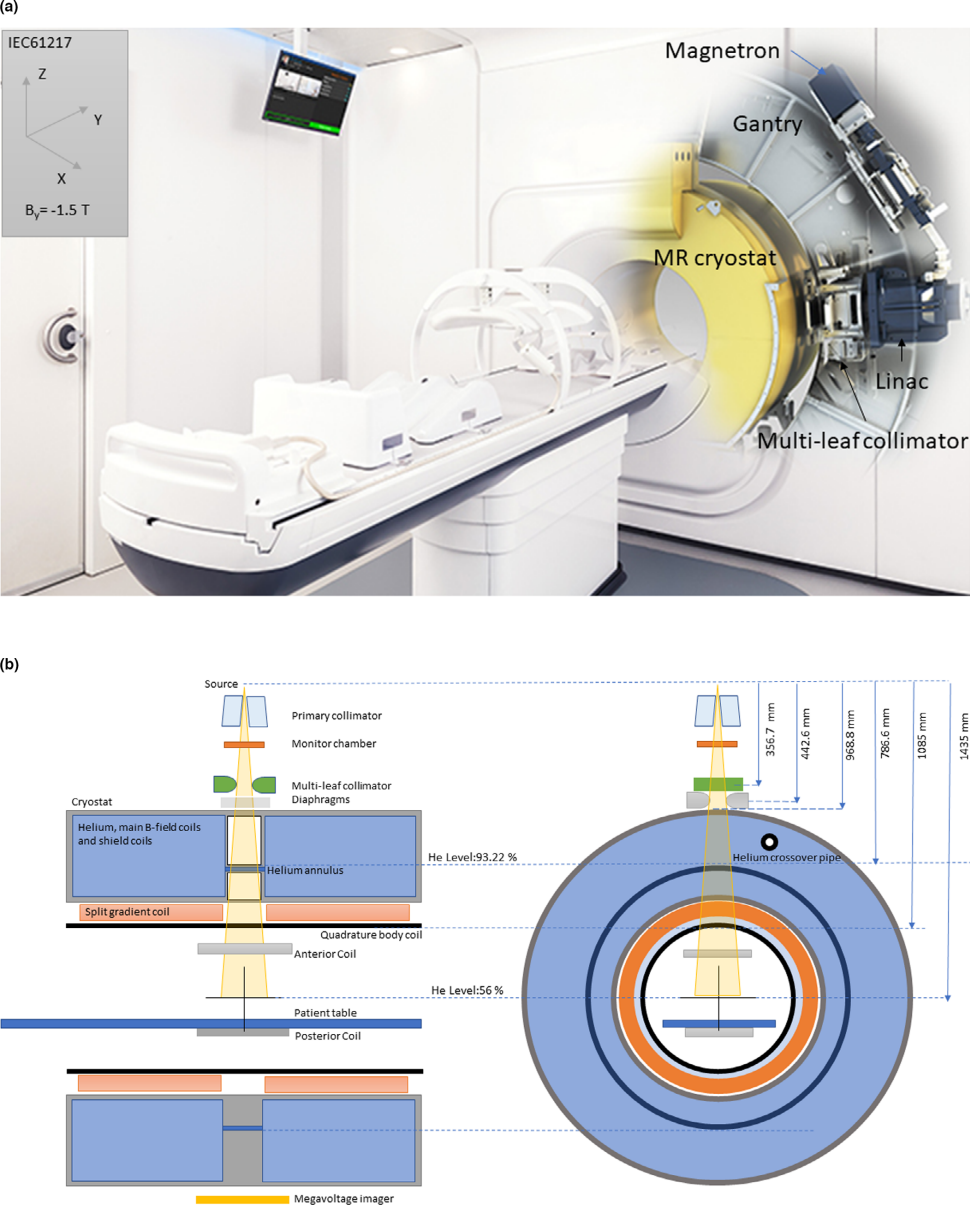
(a) Elekta Unity components, IEC61217 coordinate system, and B‐field direction. (b) Cross‐section of the beam delivery system and the magnet. The main B0 field has its vector directed out of the bore (‐ve IECY axis).

Elekta Unity (Elekta AB, Stockholm, Sweden) shown in Fig. 1 is a system integrating a 7 MV flattening filter‐free (FFF) RT system and a 1.5 Tesla (T) Philips (Philips Healthcare, Best, the Netherlands) MRI system based on the University of Utrecht concept.^5^ The system has a source axis distance (SAD) of 143.5 cm and a maximum field size of 57.4 cm × 22.0 cm with field defining diaphragms in the cross‐plane and 160 MLC leaves in the in‐plane direction, parallel to the bore of the magnet. The leaves have a nominal pitch of 0.7175 cm. A patient positioning system moves in the longitudinal direction when in the bore of the magnet, which has a 70.0 cm diameter. A terbium doped, gadolinium oxysulfide (Gd_2_O_2_S:Tb) scintillator‐based amorphous silicon megavoltage (MV) imaging panel is integrated into the system enabling machine setup, calibration, and quality assurance procedures. The imaging panel, located at 265.3 cm from the source has a pixel size of 0.04 cm and a field of view of 21.0 cm × 8.5 cm, limited by the cryostat gap separating the main MR coils that generate the static magnetic field (B0).

The system is used in combination with Elekta MOSAIQ^®^ and Elekta Monaco^®^, a Monte Carlo‐based treatment planning system that optimizes and calculates the dose distribution in the presence of a magnetic field.

### 1.2 Geometry

**Fig. 2:**
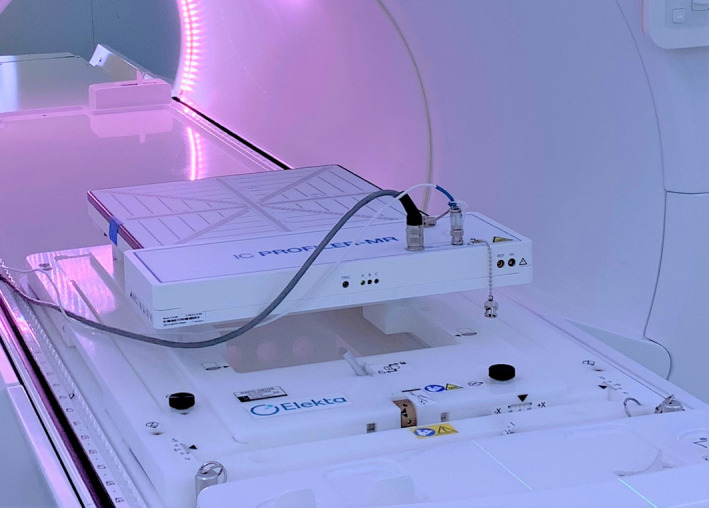
Elekta Unity QA platform attached to the table indexing with an SNC IC Profiler MR attached. Movement of the table to a mechanical end stop results in the IC profiler measurement origin being placed at isocenter (refer to section 2.4).

Both the geometry and environment of MR‐linac systems such as Elekta Unity are different to conventional radiotherapy devices. First, systems are bore type machines where the linear accelerator rotates around the MRI system. As a result, the radiation collimator is not visible in the treatment room. Unlike conventional systems, no light field or side lasers are present. This requires alternative methods of equipment alignment at the MV isocenter, for example, remote isocenter lasers, positioning of equipment using table indexing (Fig. 2), or use of MV imaging. Some restrictions in standard measurement setups may also be necessary due to bore restrictions. However, other RT equipment with bore type geometries do exist, for example, TomoTherapy^®^ (Accuray, CA, USA). Second, nonstandard source axis distances result in different beam characteristics, such as profile shape and certain penetrative quality measures, for example, dose maximum, than would be obtained at the conventional 100 cm SAD. Lastly, the beam lines of MR‐linacs may be different from conventional machines. On Elekta Unity, the beam passes through the MR cryostat, gradient coil support structure, quadrature body coil (QBC), anterior and posterior receive coils, and the patient support system. The cryostat is specifically designed to have a low attenuating, homogenous region between the superconducting coils while maximizing the B‐field homogeneity to enable diagnostic quality MR images. In this homogenous region, small variations in material thicknesses (0.01 cm) and helium level result in small variations in attenuation of the radiation beam with gantry rotation. Manufacturing tolerances of the cryostat body and QBC result in a change in beam output at isocenter for a fixed dose at the monitor chamber. The helium level also affects the attenuation. To optimize imaging performance, the superconducting magnet was designed as one system (non‐split magnet) and thus helium is present in a small annulus connecting the cryostat in the longitudinal axis through which the beam passes. The system incorporates a zero boil off MR system and as such the helium level is fixed. It is only expected to change on exceptional events, for example, a cryostat cooler fault or some service operations, including ramp up and down of the magnet. The attenuation of the liquid helium is <0.9% and thus on a change in helium level, the output of the system may change with gantry rotation when the Helium annulus contains both gaseous and liquid helium. This occurs at helium fill levels of less than 93%. During installation, the system is characterized and the attenuation of the components and helium in the beam line is determined as a function of gantry angle. This information is then used in the treatment planning system to account for the attenuation. In the case where the helium level changes after initial system characterization, the cumulative worst‐case effect for a treatment plan with multiple beam angles is less than 0.5%.

Given the above, QA tests should take the cryostat effect into account by use of a gantry‐dependent output baseline or via comparison to the treatment planning system output.

### 1.3 Effect of the MR field on radiation measurement

Due to the presence of the magnetic field, the dose distribution characteristics are different to conventional radiotherapy systems. Note that MR systems have two main magnetic field generation systems. The main static magnetic field (B0), which is always present and highly uniform (2 ppm = 3 µT RMS within an ellipsoid of 50 × 50 × 45 cm^3^) and superimposed on this is the gradient fields, which vary during MRI. In terms of dosimetry, the magnetic field can be considered uniform and the gradient field strengths neglected as these are on the order of 10’s of mT/m. Dosimetry measurements during MRI show no discernible difference to those conducted in a static magnetic field.^44^, ^45^ Thus, measurements or dose calculations performed without MRI gradient fields active are equivalent to measurements with them active, that is, the case when a patient is being imaged during treatment for motion monitoring purposes. Note that measurements of radiation during MRI require special consideration as the gradient fields and/or the RF generated by the MR system can affect the detector. This is discussed in section 2.2 but will require assessment of any dosimeter being used. The use of a nonactive detector, such as film being of preference.

**Fig. 3:**
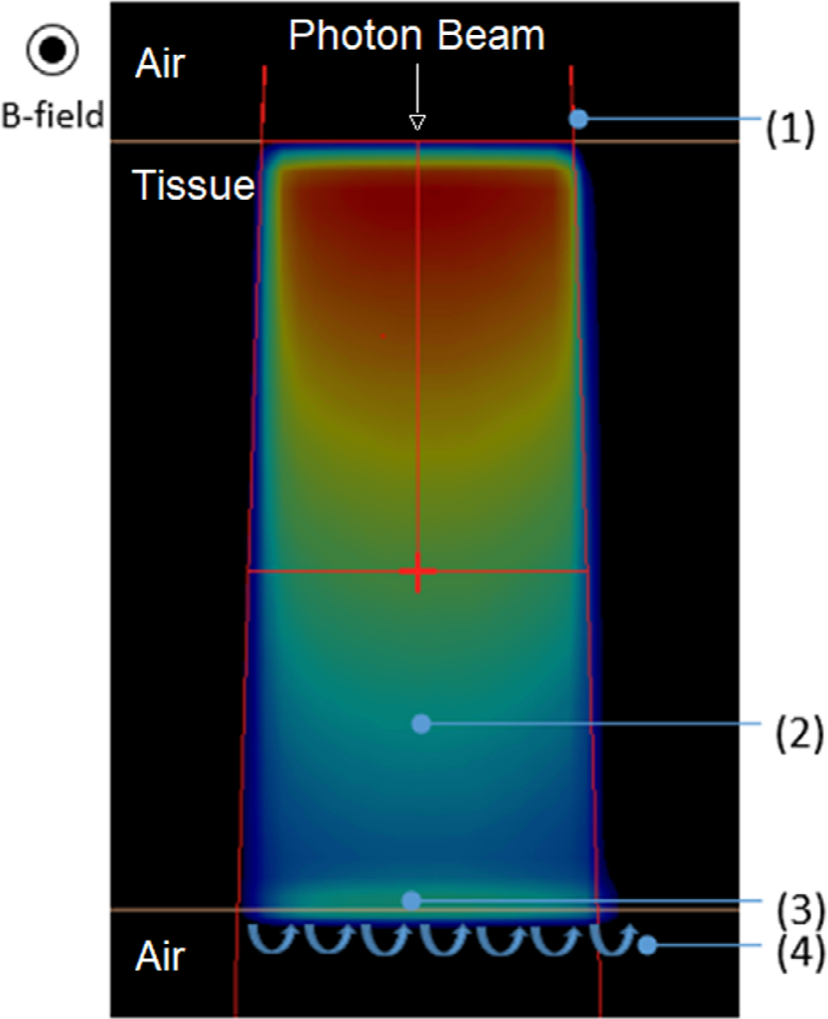
Effects on dose distribution for a 10 cm × 10 cm field in a plane perpendicular to B‐field and trajectory‐ray beam direction. 1 – Expected field edge, 2 – dose distribution with shift to the + IECX direction, 3 – Electron return effect, 4 – Electron trajectory on exiting higher to lower density material.

Fig. 3 shows the effect of the magnetic field on the dose distribution through a uniform medium and at an interface between a higher and lower density material. In a uniform medium, the dose distribution shifts in a direction that is perpendicular to the beam path and the B‐field direction. In terms of measurement, this has the following effects:

- Field size — The field “set” on the accelerator will be the dimensions of the photon fluence at isocenter (~dose distribution field size under B0 = 0 Tesla) but in the presence of the B‐field the dose distribution will be shifted.^20^, ^46^ Hence the field edges perpendicular to the photon beam and B‐field will be shifted. In this direction the central axis offset varies with depth and field size ranging from 1 mm for a 1 cm × 1 cm field at a depth of 15 mm to 1.7 mm for a 10 cm × 10 cm field at a depth of 150 mm.^24^
- Symmetry — Due to the profile shift the symmetry of the beam along the IECX axis, which is evaluated around the beam central axis, can be approximately 1% higher (for a 22 cm × 30 cm field at a depth of 10 cm) than if the magnetic field was not present. Measurement of symmetry about the field edge midpoint would yield a symmetry comparable to the case if the magnetic field was not present.
- Asymmetric penumbra — The geometric size of the penumbra on the opposing field edges perpendicular to the B‐field can vary by 1 mm for a 10 cm × 10 cm field.^22^

In addition to the above other effects can be seen at interfaces of different densities. If the electrons exit a higher density material into a lower density material, they can “return” to the higher density material, which increases the dose at the interface (Fig. 3). This effect has been termed the electron return effect (ERE) and has been investigated extensively.^46^, ^47^, ^48^, ^49^, ^50^, ^51^, ^52^, ^53^ If electrons exiting into air are not reabsorbed, they spiral along the B‐field which has been termed the electron streaming effect (ESE). This primarily occurs where there are oblique tissue angles.^54^ The effect can also occur where electrons are generated in air, on entry or exit of the patient, but this involves less electrons and is therefore a weaker effect. ESE can cause dose to be delivered to locations that are not in the treatment area in the head–foot, foot–head direction.

In all the above cases, the TPS takes the magnetic field into account during plan optimization and dose calculation.^24^, ^26^, ^55^, ^56^, ^57^ Hence it can be used to correctly calculate the dose distribution and provide the reference for comparison of expected field size, symmetry, and penumbra.

## 2 EQUIPMENT

### 2.1 Equipment overview

Measurement equipment used on conventional RT systems must be assessed for compatibility with an MR‐linac. MR compatibility falls into three categories:

- MR safe: These devices pose no known hazard in an MR environment.
- MR conditional: These items pose no known issues in a specified magnetic field under specified conditions of use.
- MR unsafe: Devices that are known to pose a hazard within the MR environment

In many cases, existing QA vendors sell specific MR/RT equipment with the intended environmental conditions of use noted. Users should however ensure their suitability for the field strength of their system and ensure that “similar” equipment, not compatible with an MR‐linac device is not used on the system. Table I provides a review of QA devices specifically designed for MR/RT systems or that have been used on such systems. In the case of Elekta Unity, the manufacturer provides tests and equipment with the system which are noted below and in section 3. In addition, Elekta Unity is provided with a QA management system, Elekta Aqua™, which serves to analyze data received from the MV imager and to record and trend results from third party equipment. Equipment such as Gafchromic film (Ashland, USA),^44^, ^58^, ^59^ water equivalent plastic, and electrometers can also be used but are not included given either their inert characteristics in a B‐field or that they are used outside the magnetic field, for example, electrometer. Where specific equipment is noted, the authors have validated them for their own use and thus users should contact the relevant device vendor and conduct commissioning tests of the equipment prior to use.

**Table I:** Example QA equipment used by members of the Elekta MR‐linac consortium on the Elekta Unity system.

QA device type	Device manufacturer/name
Daily QA	SNC Daily QA^TM^‐MR
Multi‐axis Linear detector array	PTW STARCHECK^maxi®^ MR^10^, ^13^ SNC IC Profiler™ ‐ MR^14^
2D detector array	PTW Octavius 1500 MR^40^
MV imager	Integrated MV imager^17^ (Provided with Elekta Unity)
Single detectors (Relative and reference)	***Ion chambers***
	PTW Semiflex 3D Chamber (31021)^20^, ^22^
	PTW PinPoint^®^ 3D Chamber (31022)^20^
	PTW Farmer (30013)^20^, ^22^, ^60^, ^61^, ^62^, ^63^, ^64^, ^65^, ^66^
	PTW Pinpoint^®^ Chamber (31006)^61^
	PTW Semiflex Chamber (31010)^67^
	Standard Imaging Extradin^®^ A1SL^61^, ^68^
	Standard Imaging Extradin^®^ A19 Ion Chamber^68^
	IBA Farmer type ionization chamber (FC65‐G)^22^, ^60^, ^62^, ^65^, ^66^, ^68^
	***Diodes***
	PTW microdiamond^®^ (60019)^20^, ^22^, ^23^, ^63^
	PTW Dosimetry Diode P (60016)^20^
	PTW Dosimetry Diode SRS (60018)^20^
Water tank	PTW MP1 MR Manual Water Phantom
	PTW BEAMSCAN^®^ MR^15^, ^22^
Coordinate system alignment	Elekta MR to MV phantom (Provided with Elekta Unity)
MRI QA	Philips 200 mm head phantom (Provided with Elekta Unity)
	Philips Geometric distortion phantom^32^ (Provided with Elekta Unity)
	Modus QUASAR^TM^ MRID^3D^ Geometric Distortion Analysis System
	American College of Radiology (ACR) Phantom (multiple vendors)^32^
Plan QA devices	PTW Octavius^®^ 1500^MR40^
	PTW Octavius^®^ 4D MR (pending)
	SNC ArcCheck^®^–MR^11^
	ScandiDos Delta^4^ Phantom + MR^18^
End to End	SNC StereoPHAN^TM^
	Standard Imaging Lucy
	IROC Houston phantom^42^
	Radiochromic Gels^29^, ^45^, ^69^
	CIRS STEEV
	Anthropomorphic phantoms^4^, ^41^

This table is not guaranteed to be complete and is intended to give an indication of MR/RT‐specific equipment. The reader should confirm the latest status with the relevant manufacturer. PTW (PTW, Frieberg, Germany), SNC (Sun Nuclear Corporation, Melbourne, USA), Standard Imaging (Standard Imaging, Middleton, USA), IBA (IBA‐Dosimetry, Schwarzenbruck, Germany), Modus (Modus Medical Devices Inc, London, Canada), ScandiDos (ScandiDos, Uppsala, Sweden), IROC (Imaging and radiation oncology core, Houston, USA), CIRS (CIRS,VA,USA), NE2571 (Phoenix dosimetry, Camberley, UK).

The main considerations for use of equipment on an MR‐linac are discussed in sections 2.2 to 2.4.

### 2.2 Composition

Devices may contain ferrous material that could make the device a projectile hazard — this would make it “MR unsafe.” Appropriate testing should be conducted by the user or certification should be sought from the QA device manufacturer as some ferrous content may not pose a hazard if this forms a small proportion of the overall mass of the device. Equipment packaging also needs consideration given these may not be MR safe even if the device is. In addition, “MR conditional” devices may require special considerations. This might include slow positioning of the device into the bore due to eddy currents or limitations on where and when the device should be used. In the latter case, an object placed in the bore may distort the image due to its susceptibility properties and may cause heating. These effects can be minor in plastics or can cause severe imaging artifacts in the case of metallic equipment. For electronic devices that contain metal, use of the device during MRI will either lead to image artifacts or to erroneous measurement due to electrical noise induced in the measurement device by the MR system. Some devices may be safely used for radiation measurements in a 1.5T field but may be damaged if MR imaged due to the induced currents in the device from the gradient coils or RF pulses from the system. The use of a passive detector, for example, gafchromic film^70^ should be considered if measurements are required during MRI.

### 2.3 Function and dose response of detectors

Detectors may not operate or may have their characteristics modified by the presence of a high magnetic field. In terms of active (electronic) QA devices, modifications may be required to allow them to operate correctly. In addition, radiation detectors contain materials of different densities so their measurement can be affected in the presence of a magnetic field. Specifically, the following effects can occur and need to be considered when choosing the method and detector type:

- Dose response of ionization chamber — It is well documented that the response of typical ionization chambers depends on the B‐field strength and the orientation of the chamber to the B‐field and photon beam. Correction factors need to be determined per detector type, orientation, and magnetic field strength.^19^
- Response of ionization chamber in plastic water — Air gaps between the ionization chamber and water equivalent plastic cause under or over response. This effect can be controlled by the addition of water (thus removing the air gap) around the chamber but modifications must be made to current methods and the practicality of the technique assessed.^61^, ^70^, ^71^, ^72^ The same effect can be caused by air bubbles when making measurements in a water phantom — care must be exercised to eliminate any air bubbles forming on the cap of the submerged detector.
- Additional dose shift — Depending on the ionization chamber volume, the dose distribution measured by the device might be shifted compared to the actual dose distribution, effectively resulting in a different effective point of measurement (EPOM). The EPOM shifts both laterally (in the direction parallel to the magnetic field) and vertically. The vertical movement of the EPOM is surmised to be due to electrons moving in a more lateral direction compared to the case without a B‐field and the fact that the electrons do not directly enter the detector from the top due to their modified trajectory.^20^ The authors also indicate that the lateral shifts depend on the detector material. Thus, detectors further from water equivalence have larger shifts in their EPOM. Lateral EPOM, defined as the difference in the actual central axis offset compared to the measured, ranged from −0.61 mm (0.2R_cav_) for PTW 30013 Farmer chamber to 1.01 mm for a PTW Diode P for a 10 cm x 10 cm field, indicating that large ionization chambers overmeasure the lateral offset and diodes undermeasure without any corrections applied. Thus, in the case of a beam from a gantry angle of 0 degrees, the Farmer chamber would require an offset of −0.61 mm in the IECX axis. Variations of the lateral EPOM with depth were less than 0.2 mm for a 10 cm x 10 cm field. Note that the lateral offset occurs in the direction perpendicular to the beam and magnetic field. No shift is present in the direction parallel to the magnetic field. The vertical EPOM for ionization chambers is also affected, halving for the larger ionization chambers such as PTW 30013 (B_0T_ = 2 mm (~0.5R_cav_), B_1.5T_ = 1 mm(~0.4R_cav_)) and reducing to almost zero for the PTW Pinpoint 3D. Given the authors' findings, an assessment of the effect on the chosen detector should be made by the user prior to their use.
- Profile anomalies — In the case of closely packed ion chambers within an array, a difference in average density around a chamber can cause a variable chamber response.^13^, ^14^ For QA, the relative difference to a baseline profile with the same device is unaffected. However, comparison to water tank data would require a cross‐calibration of the device to the water tank data to correct for device sensitivity and the B‐field effect on the profile measurement.
- Intentional modification of dose response of a detector — The use of a high‐density material above a detector such as film has been exploited to remove the B‐field effect on the dose distribution. One such example is to sandwich film between copper plates which has the effect of nullifying the dose shift. This technique works as the higher density copper sheets result in a reduction in electron path length which in turn results in a reduction in the effect of the Lorentz force on the electrons. Effectively this results in dose being deposited more locally to the photon interaction and without the asymmetric dose shift due to the Lorentz force. This technique has been used to measure profiles and star shot films allowing for common QA techniques to be used from conventional radiotherapy systems with only minor modifications to the methods.^73^

### 2.4 Equipment alignment

The Elekta Unity system does not require the use of conventional three axis lasers due to daily adaptive planning. Clinically a single sagittal laser is used to check patient yaw rotation relative to the table. Additionally, indexed patient positioning devices are used to approximately align the patient. Subsequent MRI and plan adaptation align the beam to the patient geometry of the day.

As most electronic QA equipment cannot be MR imaged, it is not possible to use the same approach as for a patient. On Elekta Unity, the provided solution is a table‐indexable QA Alignment Platform which is used in conjunction with the on‐gantry MV imager and a phantom containing radio‐opaque markers for calibration and alignment checks. The QA platform in Fig. 2 has interface brackets for the different radiation detectors that allow the detector to be placed reproducibly in the isocenter plane with adjustment off axis if necessary to cover larger field widths. Some phantoms and devices are also commonly positioned using the index bars and/or in‐house manufactured alignment platforms.

During machine setup, the location of the MV imager relative to the megavoltage isocenter is determined. This procedure, provided by the device manufacturer, determines the source detector distance (and hence isocenter pixel size), panel yaw rotation, and the offset of the center of the panel‐sensitive area relative to the beam central axis. Note that the offset is determined as a function of gantry angle to correct for any gravity‐related changes in panel‐source geometry. This enables the position of phantoms/platforms to be determined and for the position of system components, for example, MLC position, to be measured relative to the reference MV coordinate system.

## 3 RECOMMENDATIONS — IMAGING AND DELIVERY DEVICE QA (MACHINE QA)

Compared to a state‐of‐the‐art CBCT image‐guided radiotherapy system, machine QA for an MR‐linac device can be summarized as:

- QA requirements of a Linac, modified as required due to the machine geometry or magnetic field.
- Imaging QA of the CBCT system is replaced by comparable MR diagnostic QA with additional checks on geometric distortion.
- CBCT to MV coordinate system alignment tests are replaced by comparable tests for MR to MV.

These recommendations draw on the previous QA protocols such as AAPM TG‐142,^7^ NCS‐22,^9^ IPEM 81,^8^ and publications herein referenced and the authors of this multicenter report. It is intended as an overview of the equipment and suggested QA acceptance limits.

The frequency of the tests in this section is based on the expert opinion of clinical users and forms the basis for QA on MR‐linac systems. Across the authors institutions, daily/weekly QA (machine and MR) ranges from 45 minutes to 1 hour per day on average. Monthly QA ranges from 3 to 4.5 hours noting that some sites spread the QA over the month rather than conducting in one session. As with any new technology or treatment technique introduction, a cautious approach should be considered. As longitudinal QA results are published the frequency of the QA tests are expected to reduce leading to reduction in the cost and resource required. At the authors institutions, this is an ongoing assessment based on their experience with the system and their growing QA results database. Automation of QA tests, especially utilizing the MV imager benefit from being cost‐effective and quick to perform. As with conventional machine QA, the frequency of the tests and the action levels are to be assessed by individual institutions based on recommendations, such as this report, experience, and the clinical use of their machine. QA baselines are expected to be acquired at first commissioning of the machine, when the nominal beam parameters are changed, when the measurement equipment is calibrated or if a drift occurs between a “gold standard” reference and the measurement device. QA programs should be designed such that test equipment is regularly compared against other devices/methods. The guidance provided in the following sections highlights the use of different equipment at the various frequency levels, not only for allowing more in‐depth testing but also to allow comparison between measurement devices and techniques.

### 3.1 Recommended tests, frequency, and acceptance levels

Table II, III, IV, V list the tests, acceptance levels, and equipment recommendations for delivery device and imaging QA on an MR‐linac for daily, monthly, and annual frequencies. Compared to conventional radiotherapy QA, the only changes are the addition of the MR tests and an MRI to MV coordinate system check (MRMV). These replace the kV CBCT QA and the kV‐MV alignment check on conventional kV‐IGRT systems. Acceptance levels for tests common to both conventional and MR‐linacs are the same except for isocenter size and gantry angle which have been tightened.

**Table II:** Daily QA Tests. Suggested limits do not relate to machine performance specifications. These are provided as guidance to a “minimum” expected performance.

Test	Acceptance level	Equipment
Dosimetric		
X‐Ray output constancy	± 3%	On gantry MV detector* Ionization chamber and 1D water tank Ionization chamber and water equivalent plastic Daily QA device
Backup dose monitor	± 3%	NA
Safety		
Audio‐visual monitors	Functional	NA
CCTV check	Functional	NA
Intercom system check	Functional	NA
Equipment check (damage to PPD, Coils)	Functional	NA
Emergency stop (table) check	Functional	NA
Radiation door interlock check	Functional	NA
Radiation area monitor (if used)	Functional	NA
Beam on indicator	Functional	NA
MR Device		
National Electrical Manufacturers’ Association (NEMA) Signal to Noise Ratio (method 4)^a^	NEMA SNR method 4^a^ > 87	200 mm head phantom*
Scaling Test Transverse (TRA) and Coronal (COR)^b^	NEMA percentage difference <0.5	200 mm head phantom*
Coils and patient accessories	Check for damage	NA
Call bulb	Functional	NA

^a^ Position and size of region of interest differ to NEMA standard.

^b^ Alternating direction between measurements.

* Equipment/Software provided as part of the Elekta Unity system.

**Table III:** Weekly QA Tests. Suggested limits do not relate to machine performance specifications. These are provided as guidance to a “minimum” expected performance.

Test	Acceptance level	Equipment
Dosimetric		
X‐ray output check	± 2%	Ionization chamber and 1D tank Ionization chamber and water equivalent plastic Multi‐axis Linear or 2D detector array
Backup monitor constancy	± 2%	NA
Mechanical		
Multileaf collimator (MLC) and diaphragm positions^a^	Qualitative or quantitative (±1 mm)	On gantry detector* Gafchromic film Multi‐axis Linear or 2D detector array
Safety		
Emergency power off switched	Functional	NA
Terminate and Interlock key checks	Functional	NA
MR device		
Flood field uniformity	Elekta Unity QA acceptance levels, see Table VIIb	200 mm head phantom*
Spatial linearity		
Slice Profile		
Spatial resolution		
Magnet check (helium level)	Check level	NA
MR to MV^b^	***Translations*** ±0.5 mm to baseline ***Rotations between the MR and MV coordinate systems:*** maximum rotation for each axis: ± 0.3 degrees Mean of the absolute value of the rotations about each axis: <=0.2 degrees ***Fit*** 1 mm root mean square error (RMSE) of transform fit	MR to MV phantom*

^a^ MLC and diaphragm positions noted as weekly; however, they are commonly tested daily as part of semi‐automated MV imager‐based tests, thus consideration for alternative measurement techniques on a monthly basis can be considered.

^b^ Weekly but frequency reduction likely (see 3.4.4).

* Equipment/Software provided as part of the Elekta Unity system.

**Table IV:** Monthly QA Checks.

Test	Acceptance level	Equipment
Dosimetric		
Photon beam profile constancy	±2%	MV imager* Multi‐axis Linear detector array
X‐ray beam quality (TPR 20,10)	±1%	Ionization chamber and water equivalent plastic Ionization chamber and 1D water tank
Mechanical		
Gantry angle	±0.3^o^	Spirit level Dedicated phantom^74^
Tabletop position	±1 mm	Ball bearing at indexing location and on gantry MV imager
BLD field size check	±2 mm	MV imager* Gafchromic film 2D Detector array
Leaf position accuracy (IMRT)	±1 mm	Gafchromic Film On gantry MV imager*
Radiation isocenter size	<=0.5 mm radius	Winston‐Lutz phantom (ball bearing) with MV imager* Spoke film ‐ Gafchromic film
Safety		
Safety interlocks (Function keypad terminate key, table longitudinal, stop motors)	Functional	NA
MR device		
MR geometric test	200 mm DSV <=1 mm 300 mm DSV <=2 mm 400 mm DSV <=4 mm	Geometric distortion phantom*

* Equipment/Software provided as part of the Elekta Unity system. DSV – Diameter of Spherical Volume.

**Table V:** Annual QA Tests.

Test	Acceptance level	Equipment
Dosimetric		
X‐ray profile comparison from baseline	±1%	3D water tank and ionization chambers (s)/ microdiamond Linear detector or 2D array
Photon beam profile constancy (multiple angles)	±2%	MV imager* Multi‐axis Linear detector array
X‐ray output check	±1%	1D water tank and Ionization chamber
Spot check of field size‐dependent output factors for X‐ray	±2% for <4 cm × 4 cm, and ±1% ≥4 cm × 4 cm	3D water tank and ionization chambers (s)/ microdiamond Ionization chamber and 1D tank/water equivalent plastic
X‐ray beam quality (TPR20,10)	±1%	1D water tank and Ionization chamber
X‐ray monitor chamber linearity (output constancy)	±5% for 2 ‐ 4 MU, and ±2% for ≥5 MU.	1D water tank/water equivalent plastic and ionization chambers
X‐ray output constancy vs dose rate	±2% from baseline	Ionization chamber and water equivalent plastic Ionization chamber and 1D water tank Ionization chamber and buildup cap
X‐ray output constancy vs gantry angle	±2% from baseline	Ionization chamber and water equivalent plastic Ionization chamber and 1D water tank
X‐ray off‐axis factor constancy vs gantry angle	±2% from baseline	Ionization chamber and water equivalent plastic Ionization chamber/1D water tank
Mechanical		
Leaf position repeatability	±0.5 mm	MV imager* Gafchromic film

^a^ Use of alternative techniques to a water tank such as film or linear/2D detector arrays is envisaged as per conventional radiotherapy devices, particularly as familiarity with the MR‐linac technology increases.

* Equipment/Software provided as part of Elekta Unity.

Specific recommendations on the methods are described below with a consensus view on the optimal method and frequency. Note that while some of these tests and methods are applicable to other MR‐linac systems, these tests are described for Elekta Unity where some solutions are integrated into the system, for instance, the MV imager. Furthermore, note that other systems may require additional tests, for example, lasers and lateral table checks. In all cases, limits are based on AAPM TG‐142 or manufacturers’ guidelines. Note that the user shall assess these tests in line with their clinical practice and adjust the limits as appropriate for the technique being used. Note that the QA acceptance level is not to be interpreted as the device performance specifications. Radiotherapy systems have a tiered level of performance. Typical performance is the performance measured on a sample of machines and the device performance is that performance specification declared by the manufacturer either in the product data or performance specifications, for example, IEC60976/977. Lastly, the QA acceptance level defines when actions need to be taken by the user. These acceptance levels may be set below the device performance specifications and may be chosen by the user based on the clinical use and age of the machine. As an example, the manufacturers' device specification for dose output through the week is <1% (IEC 60976/977) whereas the QA acceptance level is 3% for daily and 2% for weekly. The latter difference allowing for different test method precision.

### 3.2 Dosimetric tests

#### 3.2.1 Dose output and backup monitor

The rigid gantry‐mounted MV imaging device can be easily used for a daily output check.^16^, ^17^ A set number of monitor units (MU) are delivered to the a‐Si based imaging panel, potentially at multiple gantry angles, to obtain a multi frame, integrated gray scale value that can be related to dose. At beam termination, a comparison can be made between dose channel 1 (MU1), dose channel 2 (MU2), and the backup MU display. While other methods for daily QA could be utilized such as daily check devices, plastic water, and water tanks, these are more time‐consuming.

Weekly or monthly absolute dose checks in the presence of magnetic field require consideration of the experimental setup and the application of specific magnetic field correction factors. Calibration of the monitor chamber should be made in water to avoid air gaps and it is recommended to calibrate 1 MU to 1 cGy at user calibration depth, at a gantry angle of 90 degrees to ensure liquid Helium is always present in the beam path. Weekly or monthly absolute checks should follow the same setup, although the use of plastic water can be considered for constancy checks. Note that the users of solid water must ensure air gaps are removed around the chamber (e.g., by adding water or gel) and measurements made in water must guard against air bubbles.^61^, ^70^, ^71^, ^72^ As yet no international standards for measurement of absolute dose in a magnetic field exist. However, application of current standards (AAPM TG‐51,^75^ NCS‐18,^76^ and IAEA TRS‐398^77^) and appropriate correction factors for the B‐field effect on measured output have been published.^19^, ^60^, ^67^, ^68^, ^78^, ^79^, ^80^, ^81^ This correction factor is dependent on chamber type (air volume) and on the orientation and direction of the chamber relative to the B‐field, as these aspects determine the trajectory of the secondary electrons in the air cavity. For the most studied chamber, PTW 300013 (PTW, Freiburg) a mean factor of 0.993 in a parallel orientation with a 1% standard deviation across experimental and Monte Carlo simulations has been reported.^60^ While any orientation and direction can be used, it is recommended the chamber axis is placed parallel to the magnetic field direction as the correction factor is smaller. The direction (location of chamber stem) has been shown to have negligible effect but it is recommended to choose a consistent direction when setting up QA procedures.

In addition, publications recommend the use of the tissue phantom ratio 20, 10 (*TPR_20,10_*) as the energy specifier as this is less affected by the magnetic field and is practically easier to measure than similar parameters in TG‐51.^79^

#### 3.2.2 Beam profile shape

For high‐frequency tests (weekly), the use of an integrated MV imager for profile deviation checks over the visible area (21.0 cm x 8.5 cm) can be used. Given that daily output checks make use of the MV imager, the beam profile shape constancy can be measured daily but must be measured at least monthly. Note that in the case of the MV imager tests, the profile measurements are relative. Profiles are measured across the images acquired and compared to a baseline image. Given that MV imagers have a flood calibration, knowledge of the actual profile shape is lost. However, for QA, the relative change from the baseline image is important. Appropriate specifications, taking into account that profile test parameters will be dependent on field size should be determined if utilizing a test field smaller than the largest.

For less frequent testing (monthly or greater), covering the clinically used field size area, tests can be conducted with gafchromic film or the use of a multi‐axis linear array placed in the bore of the magnet.

Tests at multiple gantry angles are less impactful for the MR‐linac due to a vertically mounted waveguide and thus no bending or steering magnets are present that may cause deviations with gantry rotation. Thus, measurements at cardinal angles on an annual basis are appropriate. Annual tests are expected to be conducted with film, multi‐axis ion chambers, or with 3D water tanks.

#### 3.2.3 Beam quality

Beam quality, such as the TPR_20,10_, can be measured using either a 1D water tank or water equivalent plastic, noting the additional measures required to ensure air gaps are removed. The energy measurement should be consistent with that used in the national protocol used for absolute dosimetry. More frequent energy checks can utilize the beam profile shape as a surrogate.

#### 3.2.4 Output factor

The output constancy with gantry angle and the field size output factors dependency (incl. off axis) should be checked. Both should be compared to the cryostat characterization and output factors present in the TPS beam model. Measurements can be collected with a detector in a buildup cap (for gantry angle dependency), with use of a 3D/1D water tank or water equivalent plastic for the field size output factors which can acquired at a fixed gantry angle.

#### 3.2.5 TPS baseline comparison

Measurements should use the same technique at the time of TPS commissioning and compare to the baseline taken during the commissioning phase. For the Elekta Unity system, treatment planning data collection is carried out using a 3D scanning water tank with PTW microdiamond and 3D Semiflex detectors. Use of alternative techniques for the annual QA (film or linear/2D detector arrays) are envisaged as per conventional radiotherapy devices as familiarity with the MR‐linac technology increases.

### 3.3 Mechanical

#### 3.3.1 Beam limiting device

Frequent checks of the leaves and diaphragm positions for accuracy and repeatability can be made quantitatively using the integrated MV imaging device or qualitatively using film. The weekly test may cover a reduced area and may be qualitative only, for example, using a picket fence test. Monthly tests should consider whether quantitative tests over a larger area and with gantry angle changes should be included.

**Fig. 4:**
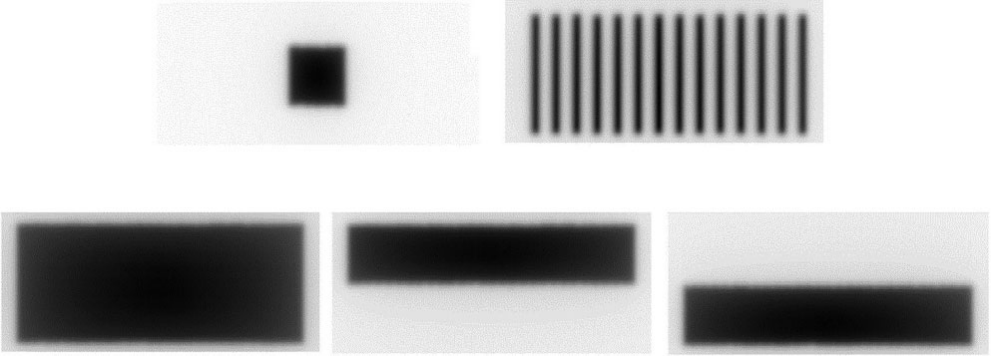
MLC and diaphragm test using Elekta Unity MV imager. A series of rectangular fields across the field of view of the imager is used to verify the accuracy of the positions for the central area (21.0 cm × 8.5 cm).

Semi‐automated routines using the on‐gantry MV imager provide the quickest check of the beam limiting device performance. It is recommended a series of rectangular fields with varying central axis offsets is irradiated to check the position of the leaves within the available field area (21.0 cm × 8.5 cm). An example test field is shown in Fig. 4 from the Elekta Aqua test, where a series of rectangular field shapes are tested. The second image in Fig. 4 shows the field used to detect the leaf positions using a field with interdigitating leaf positions. In subsequent images, leaf and diaphragm positions are then detected relative to the MV panel reference pixel using a set edge detection value which takes into account the panel response relative to water. All edges are compared to the MV panel reference pixel.

For frequencies of greater than a month and at least annually, a wider field area or changes in gantry angle should be considered. Methods including 2D arrays (subject to appropriate resolution) or film (with or without copper plates) in the bore of the machine can be used. Film tests, either using a picket fence consisting of abutting fields or a series of rectangular fields with varying offsets as in the MV imager test can be used. Note that dependent on the method, the baseline of the expected collimator positions may vary. As described previously, the B‐field results in the field edges perpendicular to the B‐field and beam direction to be shifted. Hence, the expected leaf position will be different to that displayed by the machine. Use of a technique to remove the effect, for example, copper plates, use of device not within the magnetic field, for example, on gantry MV imager, or comparison to the treatment planning system dose distribution should be considered.

For repeatability measurements, a series of repeat exposures of the same field with the collimators approaching from different directions will verify the mechanical stability of the collimator system. The use of Linac log files can also be considered to map the performance changes, taking into account that the logs are not independent of the machine.

#### 3.3.2 Gantry angle

Differences between the machine reading and measured gantry angle using a spirit level on the gantry should be recorded to stay within specifications. Alternatively the use of a phantom that can be imaged using the MV detector can be considered.^74^ The gantry angle tolerance has been tightening with respect to the conventional linac values as the gantry angle error results in increased dosimetric errors at points away from the isocenter which are more common with a bore type system. In addition, the limit also aligns with the coordinate system rotation limit in the MR to MV test.

#### 3.3.3 Table position accuracy and repeatability

Table accuracy and repeatability can be verified by attaching radioopaque markers to a table index location and using the MV detector to measure their position relative to the MV panel reference pixel or previous images. Movement of the table to different locations within the imager field of view and repeated extraction to table position 0 and repositioning to isocenter will allow assessment of the accuracy and repeatability of the table. Unlike conventional kV‐IGRT, table moves based on the fusion of the daily MR image to the planning reference image are not supported on Elekta Unity. This is because of the ability of the system to move the beam rather than the table to setup the patient in the correct location.

#### 3.3.4 Radiation isocenter

**Fig. 5:**
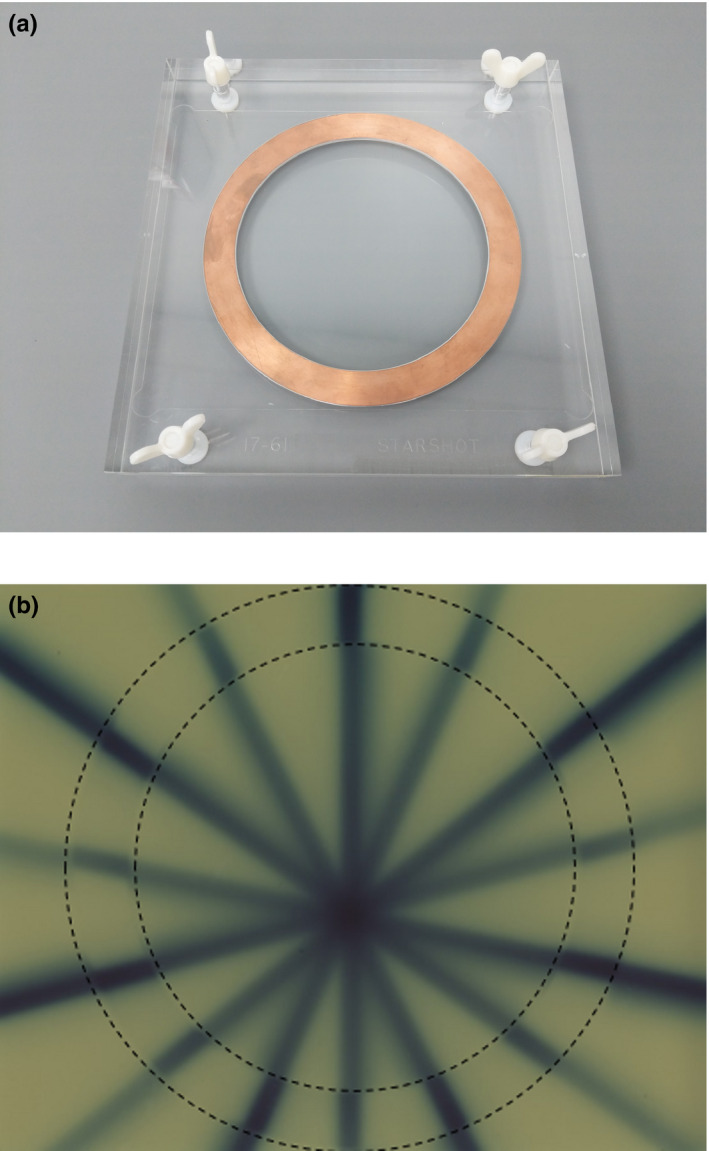
Spoke film with copper plates (a) Example Phantom and (B) Film after exposure with dotted lines showing location of copper rings.

The radiation isocenter can be measured using either a Winston‐Lutz^82^ test with a ball bearing and MV imager (provided in the Elekta Aqua software with Elekta Unity) or a spoke film. The use of a radiation field using fixed collimator components, for example, leaf sides, is advisable in the case of systems without collimator rotation. This will eliminate any systematic offsets relating from moveable collimator components. For a spoke film, the use of copper sheets^30^, ^73^ removes the dose shift caused by the magnetic field (see section 2.3). This results in the spokes effectively representing the photon beam trajectory. Fig. 5 shows an example of this equipment and test result.

The frequency of the radiation isocenter test is considered monthly because of the use of SBRT and additionally because the Unity systems include a unique beam line service system for which the isocenter size is advised to be checked after servicing. This system allows the movement of the whole beam line from the gantry structure using a swing in swing out (SISO) winch system for easy servicing and hence it is advised to check the radiation source position after the beam line is restored in addition to the other tests recommended by the manufacturer.

### 3.4 MRI

#### 3.4.1 MRI QA

The sections below detail the suggested minimum QA for the MRI device. The reader is also referred to Tijssen et al.^32^ regarding further tests for MRI system both for commissioning and QA.

##### Daily

**Fig. 6:**
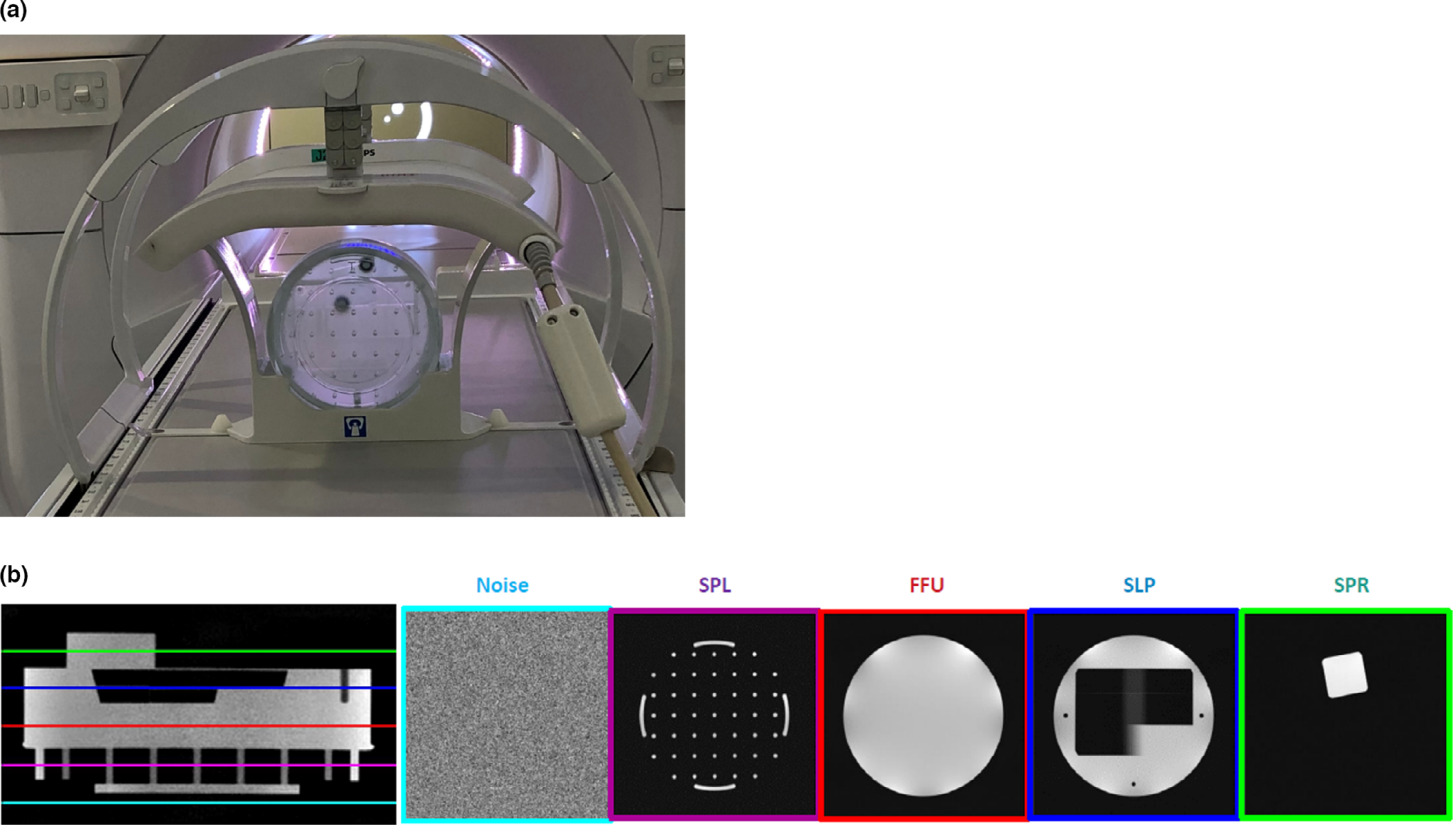
(a) 200 mm Head phantom image positioned under the Anterior Coil, (b) slices taken through the phantom image for Noise, SPatial Linearity (SPLl), Flood Field Uniformity (FFU), SLice Profile (SLP), and SPatial Resolution (SPR).

The following checks should be completed: element‐wise signal to noise ratio for the anterior and posterior coils, scaling test of the transverse and coronal planes, coils/accessory condition checks, and call bulb function check. Standard sequences (Table VI), phantom, and analysis software are provided by the manufacturer for Elekta Unity. Fig. 6 is an example of a 200 mm Philips head phantom used to assess scaling and signal to noise ratio using built‐in sequences and analysis routines. The figure shows the different sections of the phantom for noise, sPatial linearity (SPL), flood field uniformity (FFU), sLice profile (SLP), and sPatial resolution (SPR) tests. The FFU and SPL sections are used in this case for the SNR and scaling tests, respectively. The test should be performed with a fixed gantry angle for consistency and to test the influence of the MR device alone. Due to the machine design of Elekta Unity, it is not necessary to look at the gantry angle influence as this is a constant that has been taken into account in the machine design and setup. The analysis is as per the NEMA standards for magnetic resonance imaging^83^, ^84^, ^85^, ^86^ noting that the choice of region of interest placement and size may vary from the standard. The QA values here are specific to the sequences and analyses provided on the Elekta Unity system.

**Table VI:** MR Scan Protocol parameters for Daily QA tests of signal to noise ratio (SNR) and transverse and coronal scaling (Scaling T and Scaling C).

Scan name	SNR	Scaling T	Scaling C
Scan mode	2D	Multi‐slice	Multi‐slice
Scan technique	FFE	SE	SE
Orientation	‐	Transverse	Coronal
Receive coil	Anterior/Posterior	Anterior/Posterior	Anterior/Posterior
TE (ms)	4.2	30	30
TR (ms)	30	400	400
Voxel size (mm^3^)	1.3/1.3/10.0	0.98/0.98/10.0	0.98/0.98/5.0
Slice of phantom	FFU	SPL	SPL

FFE, Fast Field Echo; SE, Spin Echo; TE, Echo time; TR, Repetition time.

#### 3.4.2 Weekly

These tests include flood field uniformity, spatial linearity, slice profile, and spatial resolution. The criteria for QA passing depend on the sequence used. The tests, imaging sequences, and pass criteria for the Elekta Unity, Philips periodic image quality tests (PIQT) are given in Table VII. The phantom and software are provided with the system. The tests use the same 200 mm head phantom as the daily checks and the relevant sections of the phantom are depicted in Fig. 6b. The analysis is as per the NEMA standards for MRI,^83^, ^84^, ^85^, ^86^ noting that the choice of region of interest placement and size may vary from the standard. The QA values here are specific to the sequences and analysis provided on the Elekta Unity system.

**Table VII:** Periodic Image Quality Test (a) Sequences, FFE‐Fast Field Echo, SE‐Spin Echo, TE‐Echo time, TR‐Repetition time and (b) Test Criteria.

(a)			
Scan name	QA1	QA2	QA3
Scan mode	Multi‐slice	Multi‐slice	2D
Scan technique	SE	FFE	SE
Receive coil	MRL Ant/Post	MRL Ant/Post	Body
TE (ms)	30/100	15	3*50
TR (ms)	1000	200	1000
Voxel size (mm^3^)	1.2/1.2/5.00	1.2/1.2/5.00	0.98/0.98/15.0
Slices	Noise, SPL, FFU, SLP, SPR	SPL, FFU, SLP	FFU

(b)			
Test	Scan name	Specification	Acceptance level
Flood field uniformity	QA1 (echo 1, 2)	NEMA Signal to Noise^a^ NEMA Integral Uniformity^a^	> 59, >44 < 47, <48
	QA2	NEMA Signal to Noise^a^ NEMA Integral Uniformity^a^	>48 <47
	QA3 (echo 1, 2, 3)	NEMA Signal to Noise^a^ NEMA Integral Uniformity^a^	>45, >39, >30 <10, <10, <10
Spatial linearity	QA1	Differential linearity (35 points)	< 0.5
Slice Profile	QA1 (echo 1, 2)	NEMA Full width at half maximum (mm) NEMA Slice Integral (mm)	4.65‐5.15, 4.45‐4.95 4.85‐5.35, 4.65‐5.15
	QA2	NEMA Full width at half maximum (mm) NEMA Slice Integral (mm)	4.75‐5.25 4.9‐5.4
Spatial resolution	QA1 (echo 1, 2)	Horizontal pixel size Vertical pixel size	<1.3 mm, <1.3 mm <1.5 mm, <1.5 mm

Suggested limits do not relate to machine performance specifications. These are provided as guidance to a “minimum” expected performance.

^a^ Positioning and size of ROIs differ to NEMA standard and are specific to the 200 mm Philips phantom.

#### 3.4.3 MR Geometric distortion

**Fig. 7:**
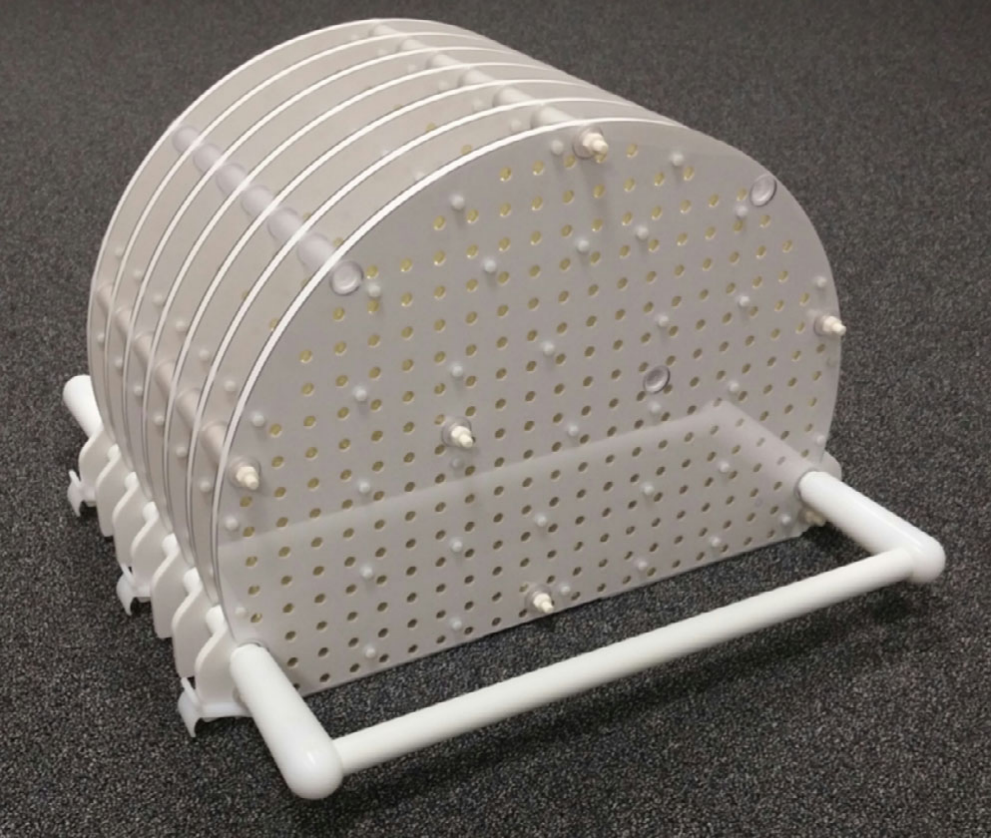
An example of a Philips MR distortion phantom as supplied with the Elekta Unity system.

The MR periodic image quality checks (PIQT), MR scaling tests, and MR to MV procedure test the small field MR geometric accuracy. In the monthly test, a phantom that measures the large field MR geometric fidelity is used. This phantom, shown in Fig. 7, with known dimensions between the MR visible markers permits the calculation of the geometric distortion. For Elekta Unity, the Philips phantom is provided with analysis software available on the MR system.

#### 3.4.4 MR to MV

**Fig. 8:**
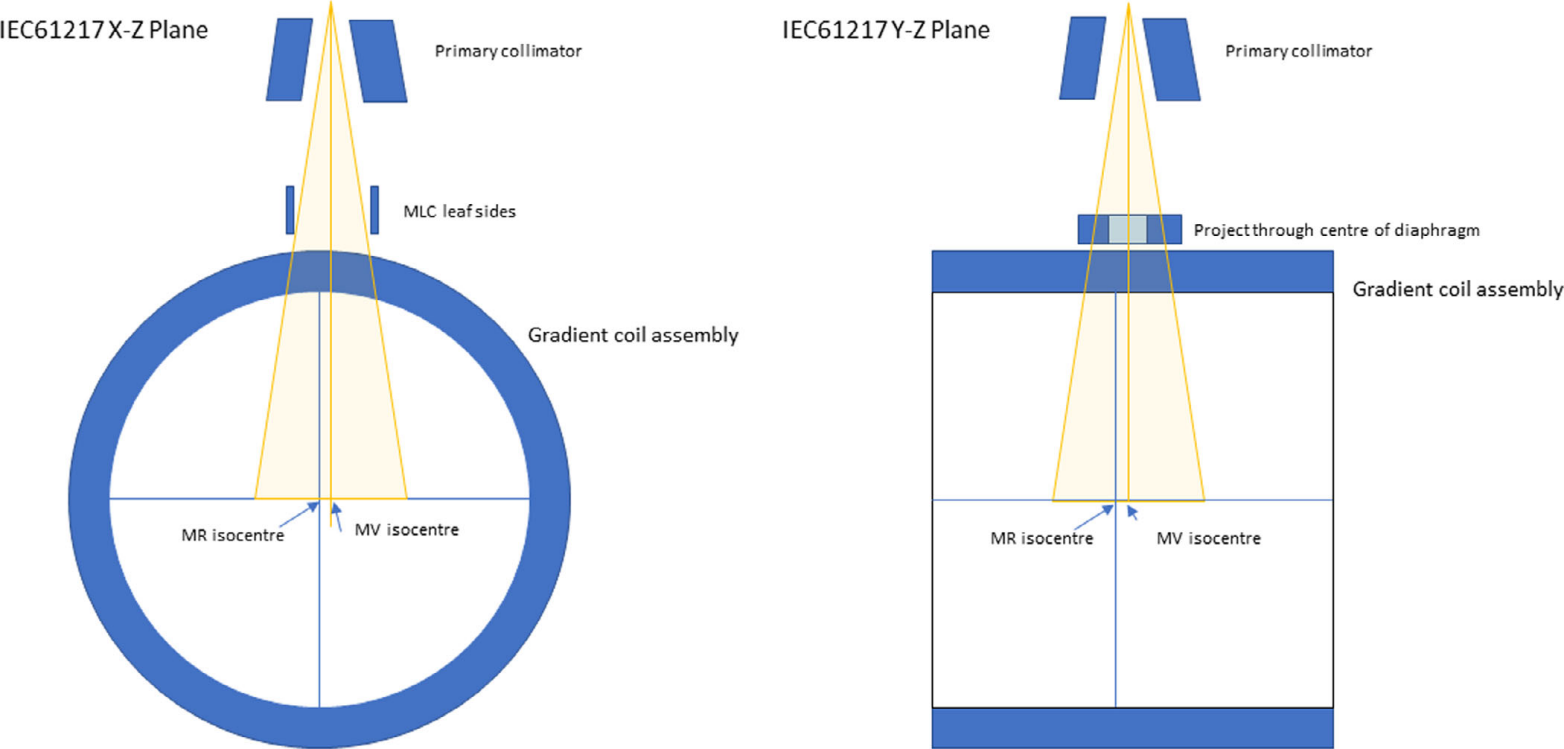
MR and MV coordinate system origins.

Analogous to the kV/MV alignment on mainstream IGRT systems, this test measures the alignment (transform) between the MR and MV coordinate systems. The MV coordinate system axes are defined by the projection of the leaf sides, the IECY axis origin by the longitudinal midpoint of the field defining x‐diaphragms, and the IECX origin as the average projection of the radiation central axis with gantry rotation. The MR system imaging center is determined by the gradient fields. Fig. 8 shows the origins of the two coordinate systems.

The purpose of the QA test is to verify that this transform has not changed due to either a MR or MV coordinate system shift or MR/MV geometric distortion. In the case of Elekta Unity, the transform is measured on machine install and applied in the treatment planning software. Specifically, all MR images are maintained within the MR coordinate system and an offset to the planning isocenter is applied prior to optimization and dose calculation. Note that only translations are applied. Rotations are not taken into account and thus limits are placed on the allowable rotation between coordinate systems.

**Fig. 9:**
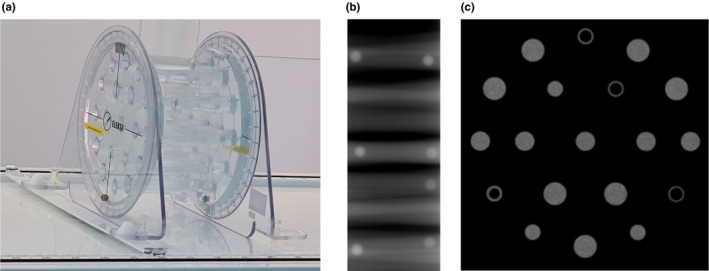
Elekta MR to MV phantom, consisting of zirconium balls surrounded by MR visible fluid to determine the alignment between the MR and MV coordinate systems, (a) MR to MV phantom, (b) X‐Ray projection, and (c) MR slice.

To determine the MR to MV transform, a phantom with markers visible in both MR and MV is used. The Elekta Unity system is provided with a phantom and software to QA the transform. Fig. 9 shows the Elekta Unity MR to MV phantom consisting of zirconium ceramic balls which are surrounded by an MR visible fluid separated by a thin layer of phantom plastic. MV projections of the zirconium balls allow the determination of their position in the MV coordinate system. A 3D MR sequence is run, and the image volume visualizes the void within the MR fluid left by the ceramic balls. This allows the determination of the position of the ball bearings in the MR geometry. Comparisons of the ball positions using template matching enable the calculation of the translation and rotation transform between the two systems. QA acceptance levels are provided for the deviation from the baseline transform, the relative rotation about all axis between coordinate systems, and the root mean square template match. Checks on the match to the template for MR and MV ball positions and the root mean square match between coordinates systems check the MR system geometry (scaling and geometric distortion) and as a secondary effect the stability of the MV imager system (position and scaling).

The test frequency is noted as weekly, consistent with current manufacturer recommendations. QA data from the system^34^ however suggests that a lower frequency check could be suitable. This is not unexpected given that a change in MR to MV transform would require either the movement of the radiation source or the MR system gradient fields. Unlike with KV/CBCT systems, where the source arm and panel could be knocked or require deployment on a regular basis, the MR‐linac has a rigid MV beam line with no adjustable bending magnets and a highly stable gradient system required for MRI.^34^ Thus, the possible failure events are much less likely and recommendations of less frequently than weekly, for example, monthly, are expected in future.

Treatment centers have also developed other phantoms that can be imaged by the two modalities^74^ and have also used the MR to MV phantom during the treatment workflow to deliver an adapted treatment to the MR to MV phantom balls and verify the position of the balls within the treatment field.^34^ In the latter case, this also verifies the correct adaption of the plan using the transform and verifies the workflow, especially if the phantom is offset from the reference plan position.

### 3.5 Checks of integrated QA equipment and supporting devices

Table VIII lists the checks that should be performed on QA equipment provided with the system. The frequency with which these devices needs testing is recommended as annual (excepting MV reference pixel), consistent with checks on other QA equipment, for example, electrometers. The equipment does not form part of the clinical workflow and hence malfunctioning equipment will result in lost QA time rather than issues with clinical treatment. Similar checks should be performed using alternative test equipment as described in the manufacturer’s documentation.

**Table VIII:** QA equipment checks.

Equipment	Test	Limit MR Linac	Equipment
MV imager	MV imaging (contrast, uniformity, noise, and spatial resolution)	Comparison to baseline for “drift” detection	Las Vegas phantom^88^
	MV imaging — scaling	1 mm	Phantom of known dimensions
	MV reference pixel	0.5 mm	Imaging of fixed machine components using MV imager.
Phantoms	Maintenance check	NA	Verify that the phantoms are not damaged and/or require a top up of the MR fluid.
Misc. Equipment			
Ferromagnetic sensor	Functional check	NA	Refer to manufacturer’s instructions
Oxygen sensor	Functional check	NA	Refer to manufacturer’s instructions

For the MV imager this is a critical piece of equipment as many of the QA tests utilize this for efficient QA. QA recommendations are made for this device below, noting that whenever it is being used for measurements should a QA failure be detected then alternative devices should be used to determine if the machine or the measurement device is in error. MV imager‐based devices have been shown to have a very stable response over time (>6 months) but may require periodic recalibration if artifacts become visible in the images.^87^ Recalibration (gain and bad pixel maps) will potentially change the output response (pixel value) of the detector and thus any QA tests reliant on this factor will need to be re‐baselined, for example,, dose output. Mechanical location of the imaging panel is important on the Elekta Unity device as the MV imaging system provides an indication of the MV isocenter.

Miscellaneous equipment includes ferromagnetic sensors and oxygen sensors which form part of the ancillary equipment for the treatment room. The manufacturers’ guidance should be followed for the methods and frequency for those devices.

#### 3.5.1 MV imaging (contrast, uniformity, noise, and spatial resolution)

An appropriate MV phantom, for example, Las Vegas, should be used to assess the contrast, uniformity, noise, and spatial resolution of the MV imager. Evaluation of the image with comparison to a reference data set to observe any failing sections of the panel.

#### 3.5.2 MV imaging — scaling and coincidence

An image of a phantom of known size and position, for example, Las Vegas, should be used to measure its dimensions against a baseline.

#### 3.5.3 MV imaging — reference pixel

The location of the isocenter projection on the panel is recommended to be checked monthly to ensure that QA measurements do not provide false negative results. The stability of the panel position can be checked by imaging fixed components of the machine, for instance, by imaging the cryostat cross over pipe and cryostat coil gap. Measurement and trending of the component edges or features of these fixed items will give an indication of imaging panel movement.

## 4 CONCLUSIONS

This report has provided an overview of the QA equipment and techniques required for measurements on MR‐linac systems particularly with a focus on the Elekta Unity system. QA on other MR‐linac systems will be comparable but will depend on machine design. A literature overview of relevant research has been reviewed and a consensus overview of typical QA measurements has been presented, in line with conventional Linac guidelines. Furthermore, an introduction to the tests and methods required on MR‐linac systems has been provided. The presented work describes a framework for comparative studies of system performance, enabling future development of QA protocols and measurement methods.

## CONFLICT OF INTEREST

David Roberts and Carlos Sandin are employees of Elekta Limited. Panu Vesanen is an employee of Philips Healthcare. All the other authors are from institutions that are part of Elekta’s MR‐linac consortium^38^ which is an international collaboration of institutions and manufacturers (Elekta, Philips) to develop the MR‐linac clinical protocols and technology.

## DATA ACCESSIBILITY STATEMENT

Data sharing not applicable to this article as no datasets were generated or analyzed during the current study.
